# Semantic interference affects speech production by increasing disfluencies, not errors

**DOI:** 10.1098/rsos.230006

**Published:** 2023-06-28

**Authors:** Kelly Rapoeye, Robert J. Hartsuiker, Aurélie Pistono

**Affiliations:** Department of Experimental Psychology, Ghent University, 9000 Gent, Belgium

**Keywords:** disfluency, connected speech, semantic interference, network task

## Abstract

Several studies have shown that different types of disfluency occur depending on the language production stage at which people experience difficulties. The current study combined a network task and a picture–word interference task to analyse whether lexical-semantic difficulty triggers errors and disfluencies in connected-speech production. The participants produced more disfluencies in the presence of a semantically related distractor word than an unrelated distractor word, while few semantic errors were made. These results support the hypothesis that difficulties at distinct stages of language production lead to distinct patterns of disfluency, with lexical-semantic difficulties leading to self-corrections and silent pauses. The results also have implications for the role of the monitoring system in connected-speech production.

## 1. Introduction

Disfluencies are phenomena that occur in all speakers during natural speech [1]. Clark & Fox Tree [2] defined disfluencies as ‘*phenomena that interrupt the flow of speech and do not add propositional content to an utterance*’ (p. 709). These phenomena occur frequently, often at a rate of 6 per 100 words [2]. However, not all speech breaks fall within the category of disfluency. For example, speakers may occasionally need to suspend their speech to signal the end of a sentence or make a statement [1]. Some disfluencies may be intentionally produced to signal a delay [2,3] or create a particular effect on speech [4]. Nonetheless, several studies have shown that different types of disfluency occur depending on the stage at which people experience difficulties in language production. For instance, some studies observed that delaying one of the earliest stages of picture naming (i.e. object recognition) resulted in increased numbers of prolongations [5, but see 6]. Studies manipulating a subsequent stage of lexical access (i.e. by comparing to-be-named objects with few or many names) found effects on numbers of self-corrections and pauses [7,8]. In this study, we will focus on the relation between disfluencies and one such level, namely lexical access.

**Figure 1.**
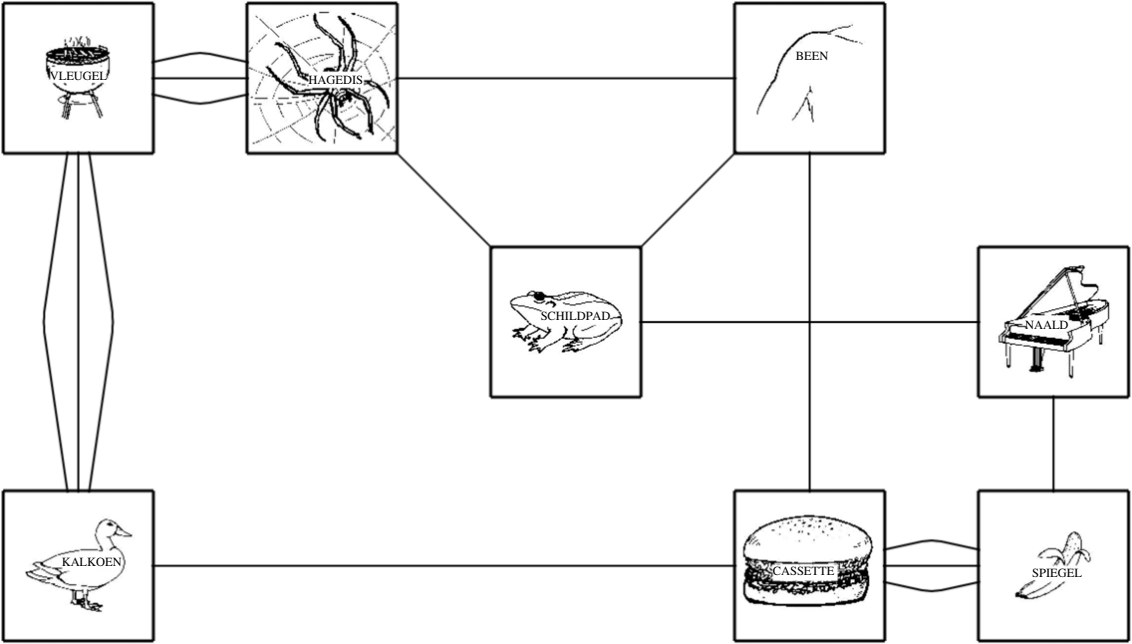
Example of a network from the current study.

Several studies have examined disfluencies related to the initial stages of lexical access (i.e. meaning-based lexical retrieval). For example, Schachter *et al*. [9] observed more filled pauses during lectures in the arts and humanities than in sciences, which was attributed to the fact that lecturers in the arts have to cope with more near-synonyms. Beattie and Butterworth [10] observed that pauses occurred more frequently before upcoming words with a low contextual probability, which suggests that these phenomena reflect ‘an act of choice’ between lexical items with similar semantic features. Schnadt & Corley [5] examined a further stage of lexical access, namely word-form encoding, via a manipulation of lexical frequency. They found more disfluencies, particularly, more prolongations, for infrequent items. However, their experiment confounded name agreement with lexical frequency. This finding makes interpretations of the underlying locus of disfluency impossible because these variables probably tap into different stages of lexical access. Nevertheless, they used a paradigm that, in principle, allows one to specifically control for the origin of disfluency in the language production system, namely, a network task [11,12]. In a network task (figure 1), participants describe a route indicated by a point marker that moves through a network of pictures. To ensure that participants speak at a relatively constant speaking rate, the point marker that moves across the network is paced at a predetermined speed, based on normal speech rate of the language under study (e.g. 42 s per network in Dutch). The network task allows for the manipulation of certain items at specific stages (e.g. lexical frequency of the pictures' names) and allows other stages to be kept constant.

In a second experiment with a network task, Schnadt & Corley [5] examined problems associated with the conceptual formulation of a speech message via visual blurring. They found that blurred pictures elicited more prolongations than did control pictures. Hartsuiker & Notebaert [7] manipulated pictures’ name agreement to induce lexical access difficulties in network descriptions. Name agreement refers to the number of different names that speakers use to refer to a given object. A low agreement reflects the activation of multiple lexical concepts and their corresponding lexical representations. Hartsuiker and Notebaert found more pauses and self-corrections before low-name agreement pictures, which was replicated in a second study [8]. One interpretation is as follows: when a picture has a low name agreement, speakers are more likely to produce errors; for example, they are more likely to start describing an object using one alternative name, such as ‘onderzeeër’ (i.e. submarine), before realizing that a another term would fit better, such as ‘duikboot’ (i.e. another word for submarines in Dutch), this change of words can be seen as a self-correction. Additionally, having issues naming a picture with low agreement is compatible with the notion that pauses represent an ‘act of choice’ between lexical words with comparable semantic properties [10]. When there is little agreement among speakers over how to label an object, choosing a lexical item becomes undoubtedly more difficult, causing pauses.

In the current study, we used a more straightforward method to analyse the effect of lexical access difficulties on disfluency production, using semantic interference. The semantic interference effect refers to the finding that picture naming typically slows down in the context of a semantically related distractor word. This effect is often observed in a picture word interference paradigm (PWI), which requires participants to name a picture while ignoring a distractor word superimposed on the image [13,14]. This effect has been largely investigated in the context of single-word production, but the implications of such interference on connected-speech production are less clear. Therefore, the current study combined two paradigms, namely the network task and PWI task. To build the network task, we used the same methods and procedure as Pistono & Hartsuiker [6,8]. In addition, we tested the effect of semantic distractor words versus unrelated distractor words. In each network, four pictures had a semantically related distractor word superimposed on them, and four pictures had an unrelated distractor word superimposed on them (figure 1). This aimed at examining the type(s) of disfluency triggered by semantic interference, and therefore, the production stage which semantic interference arguably taps into. Moreover, in order to increase the semantic interference, we accelerated the pace of the point marker moving through the network, which increased the participants' speech rate. As mentioned above, the pace of the point marker is usually set at 42 s per network in Dutch, while it was set at 35 s in the current study.

We expected more errors (i.e. distractor naming) in the semantically related condition than in the unrelated condition, as observed in single picture naming [15]. However, a substantial number of errors and disfluency was expected in both conditions, compared with previous network tasks that manipulated lexical access. Indeed, in the context of PWI and regardless of the theories that aim at explaining PWI effects (i.e. non-competitive [16] or competitive accounts [13]), authors agree on the fact that distracting information is processed and excluded from the system, which affects the time course of word production. More particularly, avoiding interference from distracting information during speech production is demanding for the monitoring system, as suggested by Dhooge and Hartsuiker [14]. The monitoring system controls the speech production process to limit errors (see [17] for a review). Some disfluencies, such as self-corrections, result from late monitoring processes (i.e. overt repairs). We therefore predicted that avoiding interference in a description task would hamper the monitoring system, leading to a substantial number of errors and disfluency overall.

Moreover, if the effect of semantic interference is similar to that of low name agreement (i.e. for which the competition mostly comes from near-synonyms, in particular in Belgian-Dutch, [18]), participants would produce more self-corrections, silent pauses and filled pauses when a semantically related rather than unrelated distractor word is superimposed on the picture. Finally, if the monitoring system adapts to the context of the task (as suggested by Dhooge and Hartsuiker), it can more efficiently adapt to the presence of distractor words over trials (e.g. less visual attention directed towards the written word), leading to less disfluency in the second half of the experiment than in the first half.

## 2. Methods

### 2.1. Participants

We recruited 42 participants (eight males, 34 females, *M*_age_ = 21.88, s.d._age_ = 3.35) via the recruiting system of Ghent University or through social media (faculty page dedicated to paid experiments). The sample size is justified in §2.4 below. They received monetary compensation of five euros for their participation in the study. All participants were naive to the purpose of the study and had either normal or corrected-to-normal vision. Participants had no diagnosed difficulties with language that could influence language production. All the participants signed an informed consent form before the start of the experiment.

### 2.2. Material and design

#### 2.2.1. Network task

A program written in PsychoPy [19] was used to create 12 networks, as in a previous studies [6,8]. Each network consisted of eight black-and-white drawings. One, two or three straight lines or curves, which were either small or large, were used to connect the images. The straight lines were horizontal, vertical or diagonal, whereas the curves were horizontal or vertical (figure 1). The point marker indicates a nine-step route through the network. In our previous experiments [6,8], the time it took the point marker to complete one network was 42 s, which was identified as the natural speech rate in Dutch. However, in the current study, we reduced the time to complete a network to 35 s in order to increase the effect of semantic interference [15].

#### 2.2.2. Stimuli

The target images and distractor words used in this experiment were the same as those of Hitomi *et al*. [20]. There were 48 black and white line drawings. Each picture (e.g. ‘dinosaurus’, ‘dinosaur’ in English) was also paired with two distractor words. One of them was semantically related to the image (e.g. ‘gorilla,’ also ‘gorilla’ in English) and the other one was unrelated to the meaning of the image (e.g. ‘lucifer’, ‘matches’ in English). Hitomi *et al*. determined the distractors' semantic similarity to each of the paired target terms using UMBC Semantic Similarity metrics (using the English translation of each word). They also ensured that the distractor words were not too strongly associated with their paired target words, using a Dutch semantic association database (Small World of Words, [21]). Furthermore, Hitomi *et al*. confirmed that distractor words were all phonologically unrelated to the target names, based on the normalized Levenshtein distance. In the current study, we also ensured that the same picture could not appear twice in the same network (i.e. with unrelated and semantic distractors). Therefore, in the first part of the experiment, half of the images had a semantically related distractor and the other half had an unrelated distractor. Pictures’ order was counterbalanced across participants.

Each network consisted of eight images, of which four had a semantic distractor and four had an unrelated distractor (figure 1). Participants were instructed to execute the task in two runs, with a break in between. Each run consisted of six networks. The pictures and the type and number of lines connecting the pictures were randomized across participants, meaning that each network was unique across the whole experiment (i.e. each participant saw 12 different networks).

### 2.3. Procedure

Each participant was individually tested in a quiet room. The experimenter provided the instructions at the start of the experiment. In the first phase, the participants completed a familiarization phase. During this phase, they were instructed to look at the computer screen and examine all pictures with their official names (i.e. without a distractor word superimposed on it). The participants were able to move to the next picture by pressing the spacebar. The familiarization phase was carried out to guarantee that disfluencies were not caused by incorrect recognition of the pictures but rather due to the presence of a distractor word.

In the second phase, participants completed three practice trials. In this phase, networks were created using target pictures and distractor words other than those used in the experimental phase, but the manipulation of semantic relatedness was similar. The experimenter provided a description of the first network to clarify the purpose and instructions, while the participants had to complete the following three networks. Feedback was provided whenever required.

The participants were asked to provide a detailed description of the network while synchronizing their explanations with the path of the dot moving through the network. A thorough description included the marker's path, shape and direction of the lines or curves, and the correct names of the pictures. Additionally, participants were told that their descriptions would be played to listeners, who would have to fill in an empty network with only the positions of the objects (i.e. empty boxes without the corresponding pictures). This was performed to elicit connected speech.

In the experimental phase, the participants completed 12 networks (with a break halfway through the experiment). Before each network, a fixation cross was displayed for 2 s. A network appeared after the fixation cross. Two seconds after the network appeared, a point marker appeared on one of the pictures. The marker traversed the network for 35 s. When it reached the end of the network, the participants had to press the space bar to continue the experiment.

### 2.4. Statistical power

In [22] a rule of thumb is proposed for mixed models in designs with repeated measures that stipulates a minimum of 1600 observations per condition. Given that an observation corresponds to the picture in the current study, an application of this formula to the current study resulted in 3200/(8 × 12) (i.e. the total number of pictures for each condition (1600 × 2 = 3200) divided by the eight images per network multiplied by the 12 networks for the entire experiment). According to this formula, 34 participants were required to ensure sufficient power. This suggests that the current experiment may be slightly overpowered, even though we anticipated the exclusion of several trials because of errors in picture naming.

### 2.5. Scoring and data analysis

Descriptions were first transcribed and coded by an experimenter, who was a native Dutch speaker. Because the manipulation focused on semantic interference, we coded only the noun phrase from any determiner up to and including the name of the picture (i.e. not the description of the paths connecting the pictures).

Disfluencies were divided into several categories: self-corrections (substitutions, additions or deletions), repetitions (sounds, syllables, words or phrases), filled pauses, silent pauses and prolongations. Because there is low agreement about the threshold that should be used to define a pause, we relied on subjective perceptual judgement and inter-rater agreement to define these phenomena, as suggested by [1]: ‘*If inter-rater reliability is built in to the annotation process, this perceptual method is probably currently the safest approach.’* [1, p. 457]. Examples of each of the disfluencies are provided in table 1, and the definitions are based on [8]. Electronic supplementary material, appendix A presents an example of a full transcript. To ensure inter-rater reliability, two further native Dutch speakers coded 24 networks each. There was 95% agreement between the coding of the experimenter and external coder 1, 87% agreement between the coding of the experimenter and external coder 2, and 85% agreement between the two external coders. The disagreement between experimenter and external coders or between both external coders was based on whether the disfluency was present or absent but also on what type of disfluency was present. Given the high agreement rates, we decided to use the experimenter's coding for all further analyses.

**Table 1.** Examples for each of the disfluencies.

category		definition	example
self-correction	substitution	when the speaker stops and continues with a word substitute	naar de het paard [/] *to the [common gender] the [neuter-gender] horse*
	deletion	when a speaker breaks off in the middle of a sentence before starting a new one	naar de [//] dan gaat hij naar boven via *to the [//] then it goes up via*
	addition	when the speaker stops before continuing with additional new information	naar de beer ijsbeer [///] *to the bear polar bear*
	other	when the speaker stops and continues with a grammatical or lexical error, or when the speaker makes a mistake with the target image	de kano de kayak [////] *the canoe the kayak (when canoe was the right target)*
repetition		repetitions of sounds, syllables, words or (part) phrases	naar de de tafel [r] *to the the table*
pause	silent pause	when the speaker delays the speech stream by being silent	naar (.) de kat *to (.) the cat*
	filled pause	when the speaker delays the speech stream by inserting a filler (e.g. uh, um)	naar uh (h) de spaghetti *to uh (h) the spaghetti*
	prolongation	when the speaker delays the speech stream by prolonging a speech sound	naar (p) de dinosaurus *to (p) the dinosaur*

We first analysed the effect of semantically related versus unrelated distractor words on all disfluency phenomena using linear mixed-effects models. The (binary) dependent variable was always the presence or absence of a disfluency. Then, to test the effect of semantically related versus unrelated distractor words on each disfluency phenomenon, we conducted generalized linear mixed-effects models with a linking logistic function. Each disfluency was treated as a binomial variable, meaning that for each picture, we coded whether it was preceded by a self-correction or not, a repetition or not, etc. A full model was constructed for each dependent variable (e.g. each disfluency), which included random effects for the subjects, items and trials. The maximal random effects structure [23] was included and reduced (by testing all possible random effect structures) until the model with the lowest Akaike information criterion (AIC) values was found [24]. To test whether participants produced more disfluencies at the beginning than at the end of the experiment, we conducted a Wilcoxon test to compare the first and second halves of the experiment. All analyses were performed using JASP [25].

## 3. Results

### 3.1. Descriptive

We excluded 1.59% (*N* = 64) of the picture naming responses because the wrong target word was produced, and 2.11% (*N* = 85) because no response was given. A wrongly produced word may correspond to a distractor name or any other word. Of the 64 errors, only 22 corresponded to distractor naming (21 distractors in the related condition and one distractor in the unrelated condition).

There was at least one disfluency in 30% of the remaining responses (*N* = 1165): 2.8% included at least one self-correction, 13.4% included at least one silent pause, 8.5% included at least one filled pause and 2.4% included at least one prolongation. Only 19 repetitions were produced in total; therefore, this type of disfluency was not analysed further.

### 3.2. Disfluency

#### 3.2.1. All disfluencies

The disfluency analysis was based only on correctly named pictures. The effect of semantically related versus unrelated distractor words was tested for disfluency (all phenomena together) using linear mixed-effects models with a random intercept for items and subjects. There was a significant effect of condition ($\chi_{1}^{2}=23.74$, *p* < 0.001, s.e. = 0.01), meaning that participants produced more disfluencies when the target picture had a semantically related distractor than when it had an unrelated distractor.

#### 3.2.2. Per disfluency category

The impact of the condition (i.e. semantically related versus unrelated distractor words) was examined independently for each disfluency type using a generalized linear mixed-effects model. Analyses based only on correctly named pictures, detailed results are provided in electronic supplementary material, appendix B.

**Figure 2.**
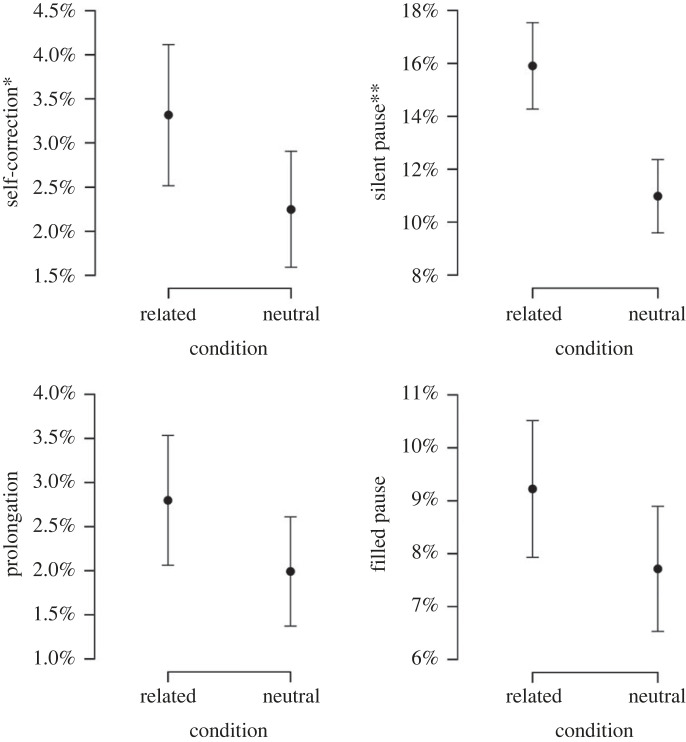
Mean proportion (and standard deviation) of pictures eliciting self-corrections, silent pauses, prolongations, and filled pauses, in the semantically related and unrelated condition. Significant results are represented on the y-axis; * < 0.05; ** < 0.01.

*Self-corrections.* Self-corrections were tested with a random intercept for items and subjects. There was a significant effect of condition ($\chi_{1}^{2}=4.18$, *p =* 0.04, s.e. = 0.10), indicating that more self-corrections were made in the semantically related condition (figure 2).

*Silent Pauses.* Silent pauses were tested using a random slope for conditions over items and subjects. There was a significant effect of condition ($\chi_{1}^{2}=8.70$, *p* = 0.003, s.e. = 0.08), indicating that more silent pauses were produced in the semantically related condition (figure 2).

*Filled Pauses.* Filled pauses were tested with a random intercept for items and subjects. We did not find a significant effect of the condition ($\chi_{1}^{2}=3.38$, *p* = 0.066, s.e. = 0.06). This indicated that there was no difference between the related and unrelated conditions (figure 2).

*Prolongations.* Prolongations were tested with a random intercept for items and subjects. This did not yield significant differences between the conditions ($\chi_{1}^{2}=2.81$, *p* = 0.094, s.e. = 0.11) (figure 2).

### 3.3. Wilcoxon signed rank test

To investigate the differences between the first and second parts of the experiment (i.e. first six networks versus last six networks), we used the Wilcoxon signed-rank test. This test revealed no significant difference in the total proportion of disfluency between the first and second halves of the experiment (*z* = 0, *p* = 0.503).

## 4. Discussion

In this study, we investigated the effect of semantic interference on connected-speech production by combining a network task with a picture–word interference task. Previous research has shown that participants are affected by semantic interference during a PWI task, leading to longer reaction times [8,13]. In the current study, we focused on the effects of semantic interference on speech errors and disfluencies. We first observed that only 22 distractor words were produced, which contradicts our predictions. Indeed, we predicted more errors (distractor naming) under the semantic condition. Even though there were numerically more errors in the semantically related condition versus the unrelated condition (i.e. respectively 21 versus 1), the small number of errors indicates that participants' monitoring system efficiently coped with the presence of distractor words to avoid errors, even under time pressure and in a situation where participants had to produce a continuous speech stream.

Nevertheless, a substantial amount of disfluency was produced in the current study compared with previous network tasks. Indeed, 30% of pictures elicited disfluency, compared with 17.9% in Hartsuiker & Notebaert [7] and 21% in Pistono & Hartsuiker [8]. This suggests that many errors were intercepted by the monitoring system, but such covert errors resulted in the production of disfluency. When all disfluencies were taken together, participants were more disfluent when a semantically related distractor word was superimposed on the target image than when it was an unrelated distractor. Therefore, we demonstrated the effects of semantic interference on disfluency production. These findings are in line with a previous study that examined disfluency production in a PWI, using time pressure as well [15]. However, this previous study was based on single picture naming rather than connected-speech production, and the proportion of disfluent answers was much lower than what we found in the current study (around 7% of disfluent trials). It therefore seems that avoiding interference is more challenging in the context of connected-speech production, which leads to more disfluencies but not more errors.

When each disfluency was analysed individually, we found that participants were more likely to produce self-corrections and silent pauses when facing a picture with a semantically related distractor word. These results are similar to those found for low-name agreement pictures [7,8]. They indicate that self-corrections and silent pauses increase in the context of semantic competition, whether this competition comes from (near) synonyms, as in a low-name agreement context, or from more distant semantic relationships (i.e. coordinates, as in PWI). As mentioned by Pistono & Hartsuiker [8], the finding that lexical access difficulties promote pauses is compatible with the claim that these phenomena reflect an ‘act of choice’ between items with similar semantic features. In addition, because of this ‘act of choice,’ speakers will be more error-prone, leading to the production of self-corrections.

Nonetheless, Pistono and Hartsuiker [8] found that lexical access difficulty induced both silent and filled pauses, whereas the production of filled pauses did not significantly increase in semantically related conditions in the current study. According to Clark *et al*. [2,3], some disfluencies, such as filled pauses, prolongations, or repetitions, are used by speakers to signal an upcoming delay in speech. In this view, these disfluencies are seen as signals (i.e. they are planned) rather than symptoms (i.e. automatic phenomena). Regarding filled pauses more precisely, different types of pauses may be used depending on the delay that is expected. Indeed, Clark & Fox Tree [2] found evidence for the hypothesis that speakers use *uh* and *um* to announce that they are initiating what they expect to be a delay before speaking. However, *uh* would indicate a minor delay while *um* would reveal a major delay. This pattern has been found in different languages: *äh* versus *ähm* in German, *uh* versus *um* in Dutch, etc. which suggests that filled pauses are controlled in part top down. In the current study, because of time pressure, participants probably had less time to signal such delays because of increased semantic competition. This could also explain why very few repetitions were produced in total. Instead, they produced disfluencies that are ‘automatic,’ namely silent pauses and self-corrections, when they encountered lexical access difficulties. Note, however, that each type of disfluency increased in the semantically related condition (figure 2), even though this was not significant for prolongations and filled pauses. Therefore, we cannot rule out the hypothesis that the lack of significant results is rather related to a lack of statistical power. Further work, using more networks in order to elicit more disfluencies, would help answer this question.

Lastly, we predicted fewer disfluencies in the second half of the experiment, but this was not confirmed in the current experiment. Indeed, a PWI task forces subjects to deal with distracted information. According to Dhooge & Hartsuiker [14], the monitoring system is required to block this type of information from the language production system, and this monitoring system is able to adapt to the context of the task. Therefore, we predicted that it would adapt to the presence of distractor words, leading to fewer disfluencies during the trials. However, this was not the case. This null result might come from the fact that only 12 networks were produced, which might not be sufficient to test this hypothesis. Finally, the current work did not consider the role of executive function in disfluency production. Indeed, performing a network task requires sustained attention, cognitive control, spatial processing, etc. that can partly account for disfluency production. Future studies should include additional executive function tasks, to examine whether they contribute to explain disfluency production. It would also be interesting to compare different manipulations of the pace of the point marker, to examine the effect it has on disfluency production. Indeed, it is possible that the difficulty of the task led participants to pay more attention to their production, leading to less errors and disfluencies than an easier task.

## 5. Conclusion

The current study combined two paradigms that are commonly used to examine the language production system, namely, the network task and the picture–word interference task. We manipulated the semantic category of distractor words to address whether semantic interference would induce more errors and disfluencies. Our results showed that participants produced few errors overall, which is why no analyses were performed on distractor naming in each condition. Furthermore, our results showed that participants produced more disfluencies in the presence of a semantically related distractor word, particularly, more self-corrections and silent pauses. This suggests that competition between semantically related items does not lead to errors in connected-speech production, even under pressure. However, it increases the occurrence of self-correction and silent pauses, two types of disfluency that are known to increase with lexical-semantic difficulties.

## Data Availability

Data, scripts and written transcripts are made available on: https://osf.io/7se5t/. The data are provided in electronic supplementary material [26].
